# O-6-methylguanine DNA methyltransferase is a favorable biomarker with proliferation suppressive potential in Breast Cancer

**DOI:** 10.7150/jca.46466

**Published:** 2020-09-01

**Authors:** Danxia Lin, Yingsheng Xiao, Binliang Huang, Xiao Wu, Chunfa Chen, Yuanke Liang, De Zeng

**Affiliations:** 1 Department of Medical Oncology, Cancer Hospital of Shantou University Medical College, No. 7 Raoping Road, Shantou 515031, PR China.; 2 Guangdong Provincial Key Laboratory of Breast Cancer Diagnosis and Treatment, No. 7 Raoping Road, Shantou 515031, PR China.; 3 Department of Thyroid Surgery, Shantou Central Hospital, No. 114 Waima Road, Shantou 515031, PR China.; 4 Department of Clinical Laboratory Medicine, Cancer Hospital of Shantou University Medical College, Shantou, PR China.; 5 Cancer Research Center, Shantou University Medical College, No. 22 Xinlin Road, Shantou 515031, PR China.; 6 The Breast Center, Cancer Hospital of Shantou University Medical College, Shantou, PR China.; 7 Department of Thyroid and Breast Surgery, the First Affiliated Hospital of Shantou University Medical College, Shantou, Guangdong, PR China.

**Keywords:** breast cancer, O^6^-methylguanine-DNA methyltransferase (MGMT), prognosis, immunohistochemistry (IHC), IRS, immunoreactive score

## Abstract

**Background:** The O^6^-methylguanine-DNA methyltransferase (MGMT) is a highly effective enzyme capable of repairing DNA damage to maintain genomic stability. Until recently, reports on the expression and potential role of MGMT in breast cancer remain controversial. This study is intended to elucidate the prognostic significance and potential function of MGMT in breast cancer.

**Materials and methods:** The immunohistochemistry assay and a series of public databases were utilized to determine the relevance between MGMT expression and clinicopathological characteristics, as well as survival outcomes in patients with breast cancer. The western blotting, qRT-PCR, proliferation, colony formation and transwell assays were used to investigate the potential function of MGMT in breast cancer cells.

**Results:** The immunohistochemistry analysis and public cancer databases exploration demonstrated that MGMT expression was significantly related to estrogen receptor (ER) positivity in breast cancer. Positive expression of MGMT predicts a longer distant-free survival (DFS) and overall survival (OS) in patients with breast cancer, especially in ER-positive tumor. The mRNA level of MGMT was significantly associated with those of ESR1, GATA3 and FOXA1 in ER-positive breast tumor. Down-regulation of MGMT expression enhanced the proliferative and invasive capacities of breast cancer cells through PTEN/AKT pathway.

**Conclusions:** MGMT is a favorable biomarker with proliferation suppressive potential in ER-positive breast cancer. Future study on targeted modulation of MGMT in the treatment of breast cancer is warranted.

## Introduction

The O^6^-methylguanine DNA methyltransferase (MGMT) is a DNA methyltransferase that exerts critical functions in DNA repair 1. It has been recognized that silencing of MGMT or a decrease in MGMT protein expression could be associated with tumorigenesis 2. For instance, aberrant promoter methylation resulting in loss of MGMT expression has been demonstrated in approximately 40% of colorectal cancers and gliomas, as well as in 25% of lymphomas and non-small cell lung carcinomas 3.

Emerging evidences have demonstrated that breast cancer is virtually a heterogeneous group of diseases with respect to molecular classification and MGMT expression 4, 5. However, reports on the protein expression profiles and prognostic significance of MGMT in breast cancer remain inconsistent. It has been shown that MGMT expression in breast tumors was up to 4-fold higher than that in normal counterparts 6, 7. S. Matsukura and colleagues reported that MGMT deficiency predicted a poor outcome in patients with breast ductal adenocarcinoma 8. On the contrary, K. Shima and colleagues demonstrated that positive expression of MGMT was correlated to shorter survival in patients with basal-like breast cancer (BLBC) 9.

These conflicting perspectives on the role of MGMT in breast cancer might stem from previous studies that evaluating heterogeneous groups of patients 10, 11. Moreover, the majority of existing studies have not presented the pertinence between MGMT expression and overall survival (OS) in patients with breast cancer 5, 8, 12, 13.

In the present study, it was found that MGMT expression was significantly associated with ER positivity in breast cancer. Moreover, MGMT could affect the proliferative and invasive capacities of breast cancer cells through regulating the PTEN/AKT pathway. These results consistently indicated that modulation of MGMT is a potential tactics in the treatment of breast cancer.

## Materials and Methods

### Cell lines and cell culture

Breast cancer cell lines including MDA-MB-231, BT-549, ZR-75-1, T-47D, and MCF-7 were purchased from American Type Culture Collection (ATCC). All the cells were maintained in Dulbecco's Modified Eagle Medium (DMEM) containing 10% fetal bovine serum (FBS) under the incubation condition of 5% CO2 humidity at 37°C.

### Small interfering RNA, Plasmids, and transfection

The pCMV empty vector and pCMV-MGMT plasmids were designed and provided by Sino Biological (Beijing, China). Small interference RNAs, as shown Table S1, were purchased from GenePharma Company (Suzhou, China). Transfection experiments were performed with addition of Lipofectamine 3000 and P3000 (Life Technology, NY, USA) by following the manufacturer's instruction.

### Patient characteristics and tissue samples

The tissue microarray (TMA) of breast cancer tissues from 275 female patients, none of which had received previous chemotherapy or other anti-tumor treatment before surgery, was collected and provided by Shanghai Outdo Biotech (Shanghai, China). Surgery took place between January 3rd, 2001 and November 1st, 2008. Patients' clinicopathological features, including gender, age, tumor size, tumor location, clinical stage and follow-up information (ending on July 30th, 2014) were incorporated for the association analysis.

### Public cancer databases

The CCLE database (https://portals.broadinstitute.org/ccle/home) was used to analyze the relative mRNA level of MGMT in a variety of breast cancer cell lines. The Breast Cancer Gene-Expression Miner v4.0 database (http://bcgenex.centregauducheau.fr/BC-GEM/GEM-requete.php) was utilized to investigate the association between mRNA levels of MGMT and clinicopathological features of BC, as well as the correlation of MGMT with ESR1, GATA3 and FOXA1.

### Western blotting assay

Cells were suspended in RIPA buffer with protease inhibitors, then the protein extracts were segregated by 10%SDS-PAGE followed by transferring onto PVDF membrane. Next, the PVDF membrane was incubated with primary antibody overnight (Table S3), and subsequently incubated for 1 hour with appropriate secondary antibodies. Finally, the electrochemiluminescence was used to detect the expression of protein.

### qRT-PCR

TRIzol reagent (Thermo Fisher Scientific, USA) was used to extract the total RNA. The PrimeScript™ RT Reagent Kit (Takara Bio Inc, Dalian, China) was used for the RNA reverse-transcription into cDNA. Primer sequences for real-time polymerase train reaction (RT-PCR) are listed and shown in Table S2.

### Cell viability assay

Cells were cultured in 96-well plates (1 x 10^3^/well). Then, 10 µl of Cell Counting Kit-8 (CCK-8) was added to each well at 0-, 2-, 3-, 4-, 5-, 6- and 7-day, with subsequent incubation for 2 hours at 37°C in an incubator. The absorbance value was detected at 450 nm.

### Transwell assay

Transfected cells (MCF-7: 4×10^3^ cells/well; MDA-MB-231: 2×10^3^ cells/well) were placed in the upper chamber in DMEM without serum, and medium containing 10% serum was added the lower chamber. After 36-hour incubation, the non-migrated cells remain on the upper of each chamber were scraped and removed with a cotton swab, and then, the migrated cells were kept intact and stained with 0.1% crystal violet. Finally, the migrated cells were counted in every five randomized fields.

### Immunohistochemistry

After deparaffinage, tissue microarrays were rehydrated in a series of graded ethanol solutions. Then, antigen retrieval was performed in EDTA buffer (1 mmol/l, pH 8.0, Boster Biological Technology Co., Ltd.) for 2.5 min. Next, the tissue microarrays were incubated with a primary anti-MGMT monoclonal antibody (Mouse, 1:50 dilution) overnight at 4°C. The slides were then exposed to 3% H2O2 for 10-20 minutes at room temperature to block endogenous peroxidase activity, with subsequent incubation with the secondary antibody for 30 minutes at 37°C. The DAB (Dako K5007 HRP/DAB+, Rabbit/Mouse) staining time lasted for 10 minutes. Hematoxylin and 1% hydrochloric acid were used for counterstaining and differentiation. The sections were then washed to blue-black in saturated lithium carbonate and sealed with neutral balsam.

The positive rate of tumor cells was categorized into an array of scores: 0: 0-5%; 1: 6-25%; 2: 26-50%; 3: 51-75%; and 4: 76-100%. The intensity of immunostaining was defined and scored as: 1 (weak or only cytoplasmic staining); 2 (moderate); and 3 (intense).

The product of the two scores was defined as the immunoreactive score (IRS), which less than 2 were defined as negative (14). The cutoff point for Ki-67 was 14%. Her-2 positive expression was defined as '+++' by IHC assay or '++' along with gene amplification detected by fluorescent in situ hybridization (FISH) of HER2/CEP17 ratio >2.0.

### Statistical analysis

The SPSS software (version 22.0) was used for all the statistical analyses in this study. Continuous variables that normally distributed are recorded as the mean ± standard error (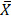
± S). The constituent ratio was reported for enumerate data. The median survival time and survival rate were demonstrated for patients' survival outcomes. Chi-square test was used to determine the correlation between MGMT expression and clinicopathological parameters.

Survival was defined as the time from surgery for breast carcinoma until the final follow-up. Breast cancer-related deaths were recorded as events for the purposes of the survival analysis. Patients who were alive at the last follow-up, or who died due to other causes, were censored. Analyses for disease-free survival (DFS) and overall survival (OS) were performed with the Kaplan-Meier method. Univariate analyses of potential prognostic factors were performed with the log-rank test. All tests were two-sided in this study and a *P* value <0.05 was considered to be statistically significant.

## Results

### MGMT positive expression was significantly correlated to ER positivity in breast cancer

**Figure 1:**
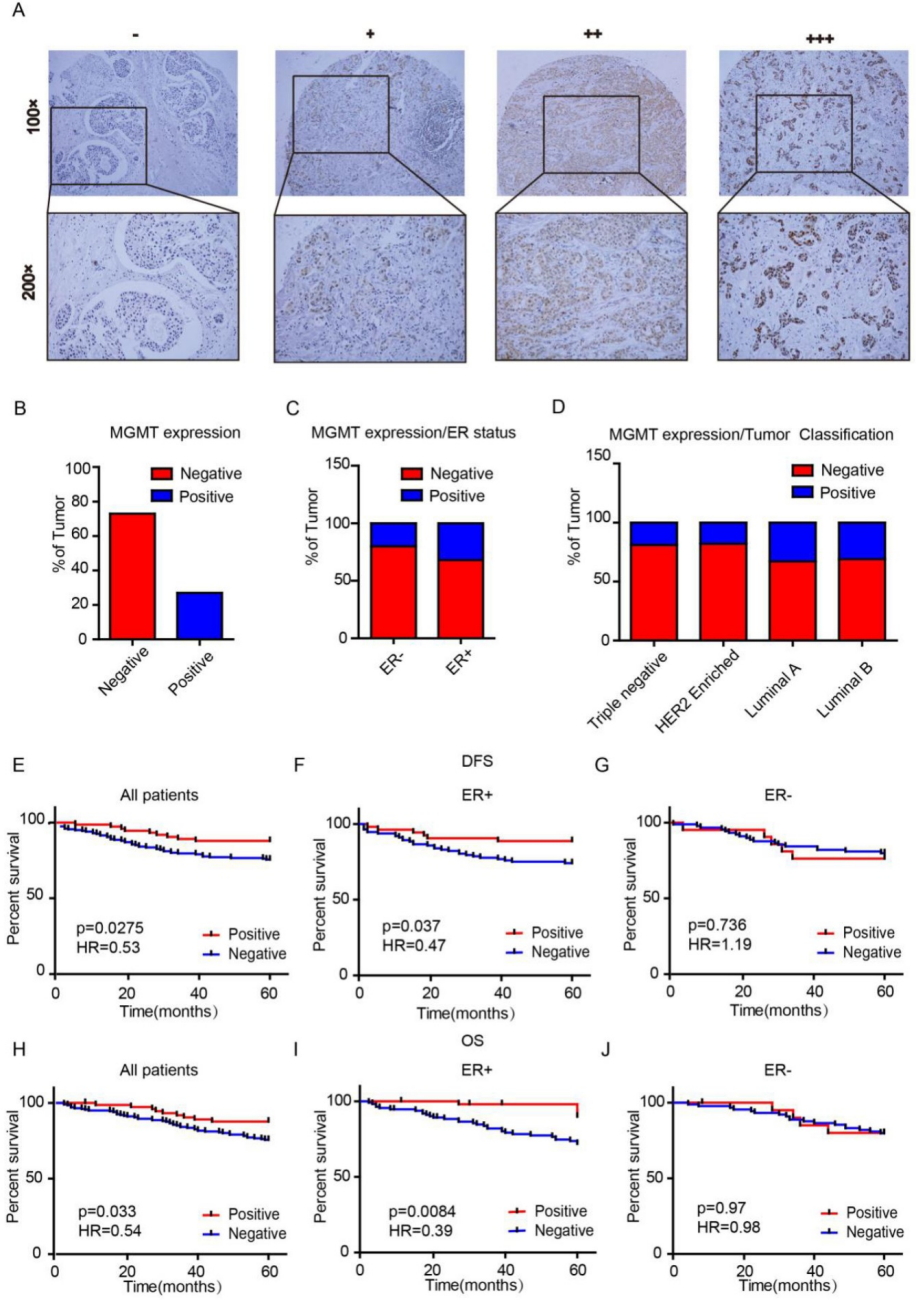
** MGMT expression is enriched in ER+ Breast Cancer** A. MGMT expression was scored as negative (-), low (+), moderate (++) or high (+++) in breast tumor tissues by using IHC. B. Percentages of total negative or positive samples from the entire cohort are shown. C and D. The TMA samples were classified according to estrogen receptor status or molecular subtype and percentage of MGMT negative versus positive. E-J. High mRNA level of MGMT was associated with longer DFS (E) or OS (H) in all BC patients and in ER+ (F and I), but not ER- BC patients (G and J).

We first assessed MGMT expression in tissue microarrays (TMAs) using IHC scored as negative (0), low (1), moderate (2) or high (3) (Fig. 1A). There were 74 (26.9%) cases with MGMT positive expression and 201 cases (73.1%) with MGMT negative expression in the 275 breast cancer samples (Fig. 1B). MGMT positivity in the ER-positive group was markedly higher than in the negative group (*p=*0.023). None of the other clinicopathological parameters analyzed, including age, lymph node metastasis, primary tumor location, tumor grade, tumor size, TNM stage, progesterone receptor expression, Her-2 or Ki-67, were significantly associated with MGMT expression (*p* >0.05, Table 1). The positive rate of MGMT in ER+ breast cancer tissues (32%) was significantly higher than in ER- counterparts (20%) (Fig. 1C). Moreover, the positive rate of MGMT was significantly higher detected in Luminal A (33%) and Luminal B (31%) subtypes than HER-2 (18%) and Basal-like (19%) subtypes (Fig. 1D).

Univariate analysis demonstrated that the 5-year DFS rates were 87.8% and 75.6% (H*R=*0.53, *p=*0.0275) in the MGMT-positive and -negative groups, respectively (Fig 1E). The 5-year overall survival (OS) rates were 89% and 74% (H*R=*0.54, *p=*0.033) in the MGMT-positive and -negative groups, respectively (Fig. 1H). Subgroup analyses indicated that high mRNA expression of MGMT was significantly correlated to longer survival in ER positive- (DFS: H*R=*0.47, *p=*0.037 OS: H*R=*0.39, *p=*0.0084) (Fig. 1F and 1I), but not in ER negative- BC (DFS: H*R=*1.19, *p=*0.736 OS: H*R=*0.98, *p=*0.97) (Fig. 1G and 1J).

### MGMT is particularly high expressed in ER-positive breast tumor and associated with the expression of ESR1, GATA3 and FOXA1

**Figure 2:**
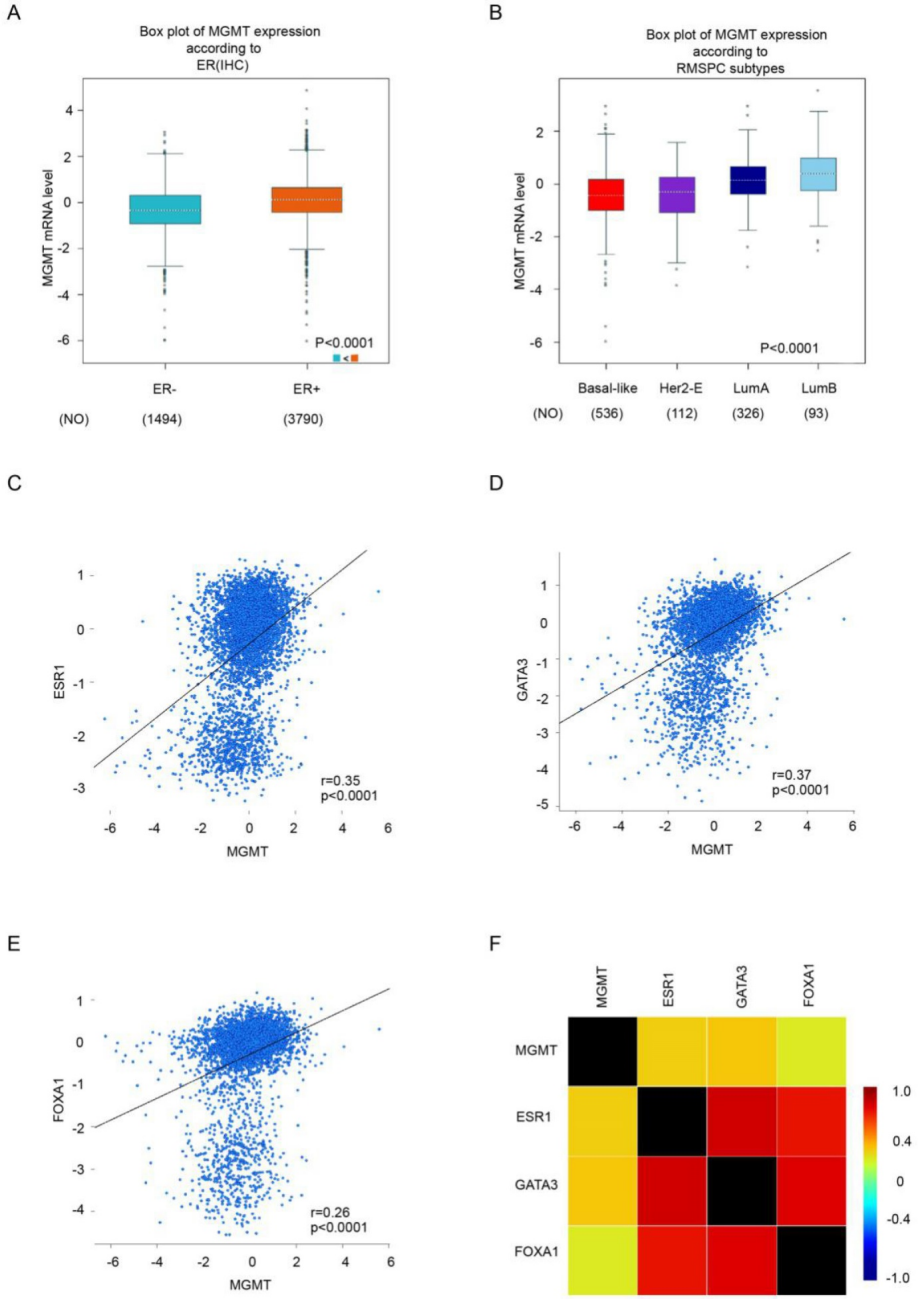
** Higher expression of MGMT correlated with high expression of ESR1, GATA3 and FoxA1.**A. The mRNA expression level of MGMT in BC patients with ER (-) and ER (+). B. The expression of MGMT in several subtypes of breast cancer patients. C. Gene correlation targeted analysis between MGMT and ESR1. D. Gene correlation targeted analysis between MGMT and GATA3. E. Gene correlation targeted analysis between MGMT and FOXA1. F. Correlation map for all patients among MGMT, ESR1, GATA3 and FOXA1.

Through analysis in bc-GenExMiner v4.0 database, we found that the mRNA level of MGMT in ER positive subgroup were significantly higher than ER negative subgroup of breast carcinoma (Fig. 2A). Moreover, the highest MGMT expression was observed in Luminal subtypes of BC (Fig. 2B), all of the group comparisons were shown in Table S4.

Gene correlation targeted analysis revealed that higher mRNA level of MGMT was associated with higher mRNA level of ESR1 (*r=*0.35, *p* <0.001) (Fig 2C), GATA3 (*r=*0.37, *p* <0.001) (Fig 2D) and FOXA1(*r=*0.26, *p* <0.001) (Fig. 2E). Correlation maps for all patients among MGMT, ESR1, GATA3 and FOXA1 were showed (Fig. 2F). These results demonstrated the level of MGMT, primarily high expressed in ER-positive breast tumor, was positively associated with ESR1, GATA3 and FOXA1.

### MGMT modulates the activity of PTEN/AKT pathway

**Figure 3:**
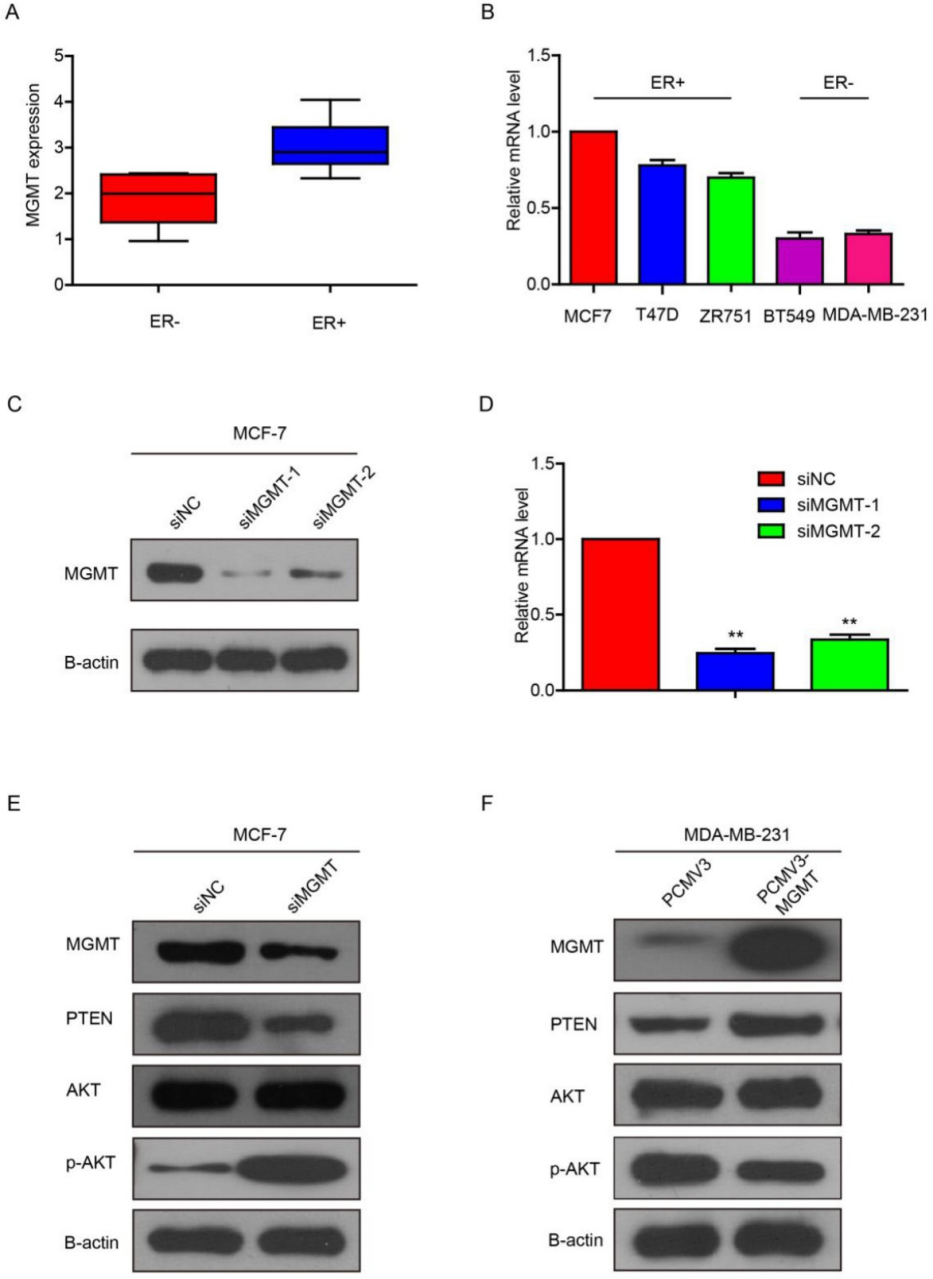
** MGMT can modulate the activity of PTEN/AKT pathway**A. MGMT expression in ER+ or ER- status of breast cancer cells using CCLE analysis. B. MGMT expression levels in various human breast cancer cell lines were tested via qRT-PCR. C and D. MCF-7 cells were transfected with two different sequences of MGMT siRNAs. E. Protein levels of MGMT, PTEN, AKT and p-AKT were detected by Western-blot analysis in siMGMT transfected MCF-7 cells, PCMV-MGMT transfected MDA-MB-231 and their control cells.

In the CCLE public database, we analyzed the expression profile of MGMT in a series of cancer cell lines. It was showed that MGMT expression in ER-positive breast cancer cells was significantly higher than in ER-negative counterparts (Fig. 3A). Next, we performed qRT-PCR assays and found that MGMT was highly expressed in ER-positive breast cancer cell lines, including MCF-7, T-47D and ZR-75-1. In contrast, MGMT expressed a significantly lower level in ER-negative breast cancer cell lines, including MDA-MB-231, BT-549 (Fig. 3B).

Next, we knocked down MGMT via transfection with siMGMT in MCF-7 cells. It was found that the MGMT protein and mRNA expression were successfully down-regulated, as shown in Fig. 3C and 3D. Because siMGMT-1 was more efficiently than siMGMT-2 in knocking-down MGMT, it was used as the main sequence for the following experiments. After transfection with siMGMT in MCF-7 cells, the expression of PTEN decreased, while the expression of pAKT (Ser473), but not total AKT, drastically increased (Fig. 3E). After transfection with pCMV-MGMT plasmid in MDA-MB-231 cells, the protein level of PTEN increased, while p-AKT (Ser473) expression declined accordingly (Fig. 3F). These results indicated that MGMT was able to regulate the PTEN/AKT pathway.

### MGMT suppresses the proliferation and motility of breast cancer cells

**Figure 4:**
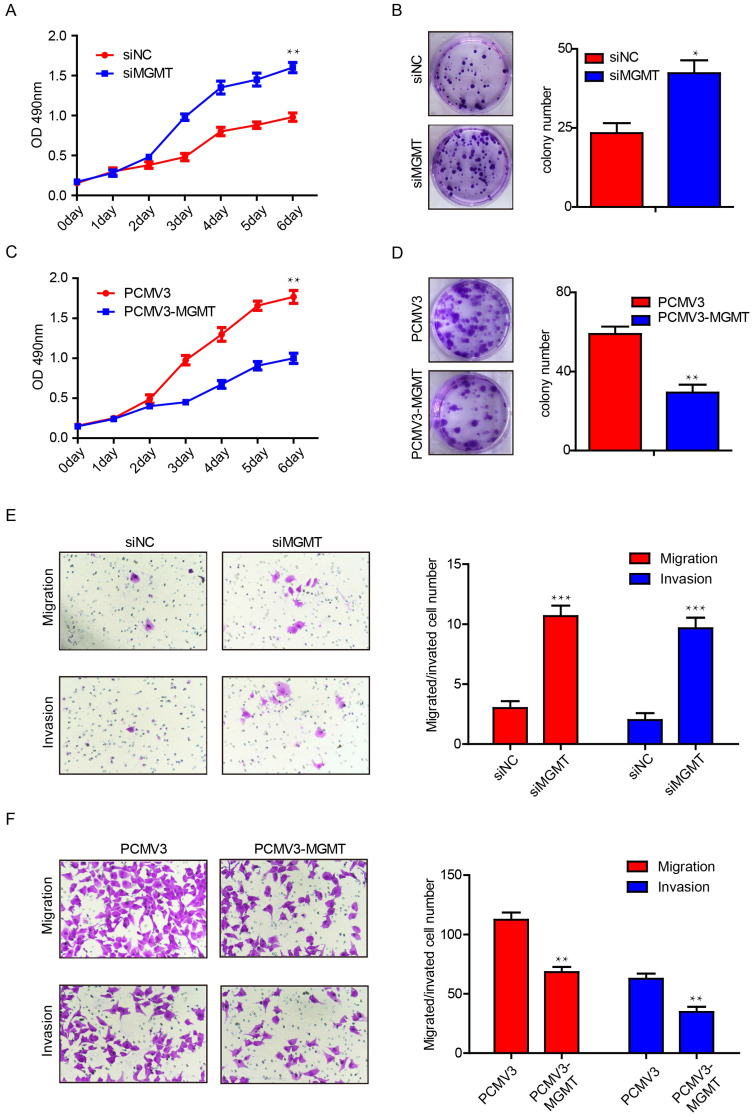
** MGMT expression could suppress the cell proliferation and migration in breast cancer cells.** A and B. Inhibition of MGMT could enhance cell growth and colony formation in MCF-7 cells. C and D. Overexpression of MGMT can suppress cell growth and colony formation in MDA-MB-231 cells. E. The cell migration and invasion ability was evaluated using a transwell assay in MCF-7 cells transferred with siMGMT and its control. F. The cell migration and invasion ability was evaluated using a transwell assay in MDA-MB-231 cells transferred with PCMV-MGMT and its control.

To further investigate the potential function of MGMT in breast cancer, we performed proliferation and colony formation assays in MCF-7 and MDA-MB-231 cells. It was found that suppression of MGMT was able to promote cell growth by 1.5-fold and increase the number of colonies by 2-fold in MCF-7 cells (Fig. 4A and 4B). In contrast, MGMT overexpression could suppress cell proliferation by roughly 2-fold and decreased the number of colonies by 2-fold in MDA-MB-231 cells (Fig. 4C and 4D). Next, transwell assays were conducted to evaluate the impact of MGMT on the migratory capacity of breast cancer cells. Suppression of MGMT was able to promote cell migration and invasion in MCF-7 cells (Fig. 4E), while overexpression of MGMT resulted in inhibition of the migration and invasion of MDA-MB-231 cells (Fig. 4F). These results revealed that MGMT exerted a pivotal role in regulating the proliferative and migratory abilities of breast cancer cells.

**Figure 5:**
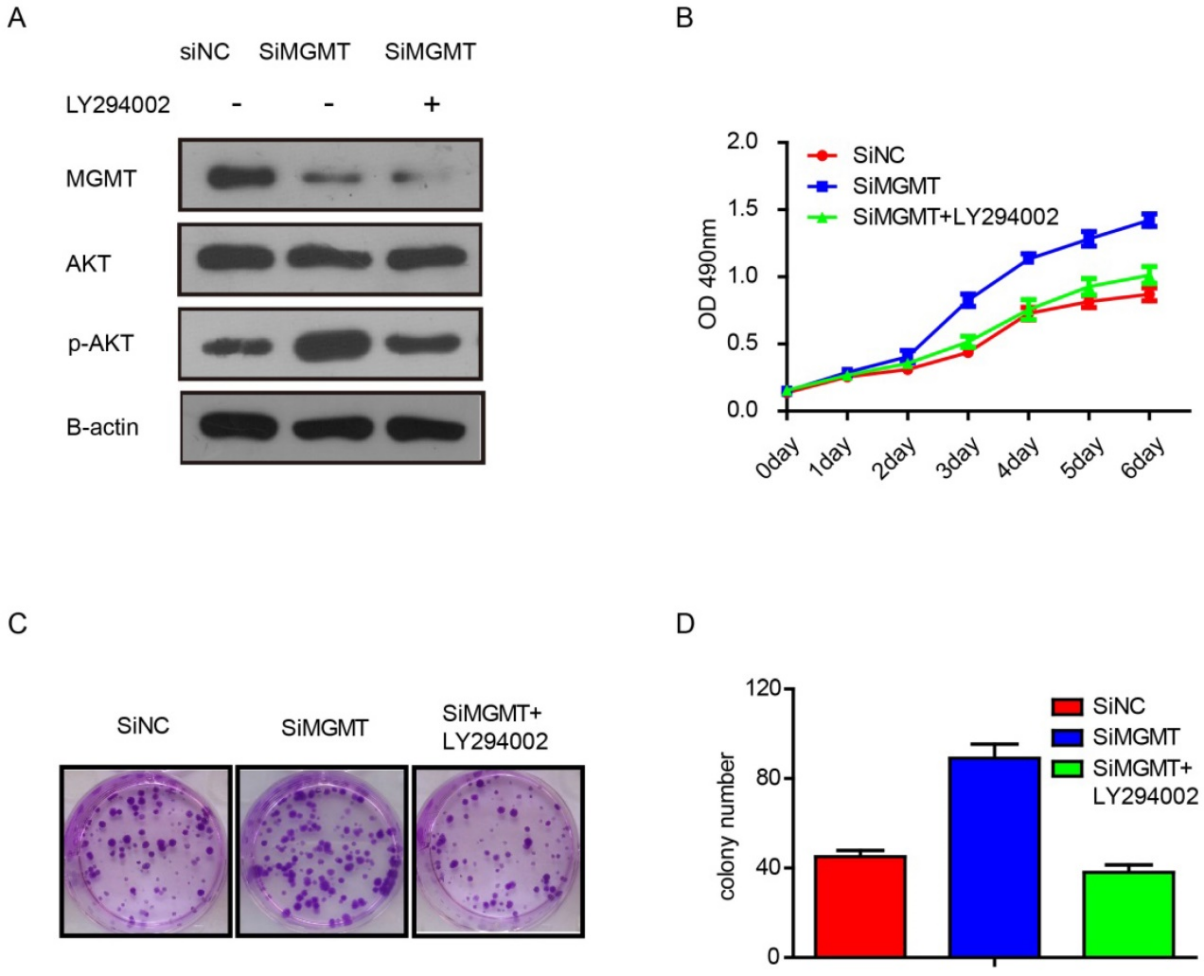
** The inhibitor of the AKT phosphorylation (LY294002) can weaken the effects of MGMT on the PI3K/AKT pathway.** A. The protein levels of MGMT, AKT and p-AKT were detected by Western-blot analysis in siMGMT transfected MCF-7 cells, with addition of LY294002 and their control cells. B-D. The cell growth curves and colony formation of MCF-7 cells transfected with siMGMT or siMGMT with LY294002.

To verify whether the effects of MGMT are dependent on the PI3K/AKT pathway, we performed additional experiments with inhibitor of AKT phosphorylation, As shown in Figure 5, it was found that knocking down MGMT could increase expression of p-AKT, but addition of the AKT phosphorylation inhibitor (LY294002) eliminated this effect (Fig. 5A). What's more, suppression of MGMT was able to promote cell growth and increase the number of colonies in MCF-7 cells. The LY294002 also can weaken the effects of MGMT (Fig. 5B-D).

## Discussion

The O^6^-methylguanine-DNA methyltransferase (MGMT) is a DNA repair enzyme capable of dissociating alkyl adducts from the O^6^ position of guanine, thereby providing an essential defense mechanism for the normal cells against malignant transformation 14. Expression of MGMT protein is discrepant in normal and tumor tissues and has not been well characterized across different tumor types 15, 16. A number of studies have demonstrated that MGMT levels were higher in tumors as compared to adjacent normal counterparts 17. A study of patients with oral squamous cell carcinoma reported by Sawhney and colleagues found a gradual loss of expression of MGMT during the transition from hyperplasia to dysplasia, suggesting that a diminution in MGMT expression might be a critical step in oral tumorigenesis 16.

Promoter methylation of MGMT is a critical mechanism of MGMT silencing, resulting in the loss of MGMT expression or MGMT negativity 18. A meta-analysis by Nairui and colleagues suggested that MGMT promoter methylation was significantly associated with decreased expression of MGMT protein and a lack of estrogen receptor (ER) expression 15. In the present study, MGMT expression was also found to be significantly associated with ER positivity in breast cancer. However, the expression of MGMT was not associated other clinicopathological parameters, including age, lymph node status, tumor location, tumor size, PR status, Ki-67 index and Her-2 status.

Survival analysis demonstrated that MGMT positive expression was associated with a better DFS and OS in patients with breast cancer. This result was consistent with the study by Cayre and colleagues, indicating that low MGMT expression was significantly related to poor survival 5. MGMT negativity resulting from methylation of MGMT may contribute to alkylating agent sensitivity in breast cancer, thereby leading to a longer DFS for patients receiving postoperative adjuvant chemotherapy 19. Moreover, we found that MGMT gene was positively associated with the classical luminal epithelial regulation genes, including ESR1, GATA3 and FoxA1. It is, therefore, postulated that MGMT might act as a potential modulator of breast epithelial phenotype and a pivotal prognostic biomarker for patients with BC.

In addition, we found that silencing of MGMT was able to inhibit the PTEN expression and increase the p-AKT level, while overexpression of MGMT could increase the PTEN expression and decrease the p-AKT level. These findings confirm the perspective of Neto's study, which suggested that there was a positive correlation between the expressions of MGMT and PTEN 13. Moreover, Zhang, L. H. and colleagues have reported that TRIM24 can regulate MGMT expression through PI3K/Akt/NF-κB signaling transduction 20. We hypothesized that MGMT can negatively regulate Akt via up-regulation of PTEN in breast cancer.

MGMT has been reported to be associated with tumor growth and metastasis in patients with glioblastoma and cholangiocarcinoma. Chen, J. and colleagues have found that knockdown of MGMT induced cell cycle entry by down-regulating p21, p27, and Cyclin E expression, thus promoting ICC proliferation 21, 22. A study from Li, C. et al. showed that silencing of MGMT increased the invasive and metastatic potentials of glioma cells via up-regulating MMP2 23. However, the role of MGMT in breast cancer was poorly elucidated. Our study demonstrated that silencing of MGMT could promote the proliferative and invasive capacities of MCF-7 cells. MGMT overexpression was able to suppress the proliferative and invasive capacities of MDA-MB-231 cells.

**Figure 6:**
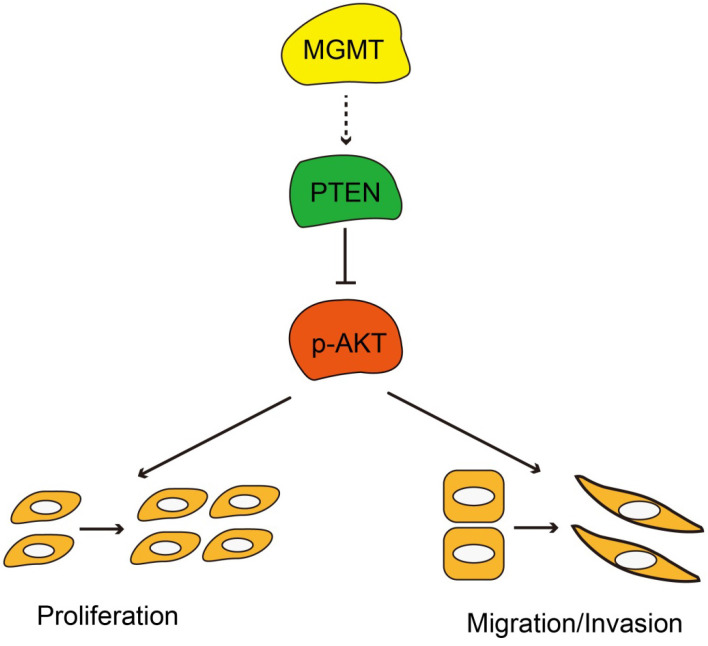
Schematic diagram showing MGMT suppressed the activity of AKT pathway via up-regulation of PTEN, thus suppressing proliferation and migration / invasion.

In summary, MGMT expression was significantly associated with ER positivity in breast cancer. Positive expression of MGMT predicted a better DFS and OS in patients with breast cancer, especially in ER-positive breast cancer. Moreover, MGMT expression could affect the proliferative and invasive abilities of breast cancer cells through the PTEN/AKT pathways (Fig. 6). Future studies on the specific role of MGMT in the pathogenesis and development of breast cancer, particularly in ER-positive subtype, are warranted.

## Supplementary Material

Supplementary tables.

## Figures and Tables

**Table 1:** Correlation between clinicopathologic parameters and expression of MGMT in breast cancer (n = 275)

Clinicopathologic parameters	n	MGMT		*P* value
		Negative	Positive	
**Age**				0.398
<60	171	128	43	
≥60	104	73	31	
**Lymph node**				0.709
Negative	124	92	32	
Positive	151	109	42	
**Location**				0.056
Left	120	94	26	
Right	155	107	48	
**Grade**				0.959
I-II	205	150	55	
III	70	51	19	
**Tumor size**				
T0-T2	246	178	68	0.425
T3-T4	29	23	6	
**Staging**				
I-II	192	143	49	0.259
III	83	58	25	
**ER**				
Negative	110	88	22	0.023^#^
Positive	165	113	52	
**PR**				0.202
Negative	147	111	36	
Positive	128	90	38	
**HER2**				0.499
Negative	195	143	52	
Positive	80	58	22	
**Ki67**				0.244
Low	171	122	49	
High	104	79	25	

^#^ *p*<0.05.

## Abbreviations

IHC: immunohistochemistry
MGMT: O^6^-methylguanine-DNA methyltransferase
TMA: tissue microarray
FISH: fluorescent in situ hybridization
Her-2: human epidermal growth factor receptor-2
ER: estrogen receptor
PR: progesterone receptor
BLBC: basal-like breast cancer
DFS: disease-free survival
OS: survival overall
