# Supplementary material for: O-6-methylguanine DNA methyltransferase is a favorable biomarker with proliferation suppressive potential in Breast Cancer
Source: J Cancer. 2020 Sep 1;11(21):6326–36. doi: 10.7150/jca.46466 (PMC7532496; doi:10.7150/jca.46466)
Supplement: Supplementary file 1 — Supplementary tables. [file jcav11p6326s1.pdf]

Supplementary

Table S1. Oligonucleotide sequences for siRNA constructs

Small interference RNAs	sense（5'-3'）
siMGMT-1	5′- AAGCTGGAGCTGTCTGGTTGT - 3″
siMGMT-2	5′- CCAGACAGGTGTTATGGAA - 3′

Table S2. Primers used in real-time PCR

Gene	Forward primer	Reverse primer
MGMT	5′- ATGGAT GTTTGAGCGACACA -3′	5′- ATAGAGCAAGGGCAG CGTTA -3′
PTEN	5′- CAGAAAGACTTGAAGCGTAT -3′	5′- AACGGCTGAGGGAACCTC -3′
B-actin	5′- AGCGAGCATCCCCCAAAGTT -3′	5′- GGGCACGAAGGCTCATCATT -3′

Table S3. Antibodies used in this study

Antibody	Cat. #	Company	Con.	Species
Anti-MGMT	AF0228	ZhongshanJinqiao（Beijing,China)	1:2000	Rabbit
Anti-PTEN	ab170941	Abcam (Danvers,MA,USA)	1:1000	Rabbit
Anti-AKT	C67E7	Cell Signaling Technology (Danvers,MA,USA)	1:1000	Rabbit
Anti-p-AKT	D9E	Cell Signaling Technology (Danvers,MA,USA)	1:1000	Rabbit
Anti- B-actin	8H10D10	Cell Signaling Technology (Danvers,MA,USA)	1:3000	Mouse

Table S4: The results of Dunnett-Tukey-Kramer’s test for pairwise comparison in different molecular subtypes of breast cancer.

mRNA	Pairwise comparison of molecular subtypes	P value
MGMT	LumA>Basal	< 0.0001
	LumA> HER2	< 0.0001
	LumB>Basal	< 0.0001
	LumB> HER2	< 0.0001
	LumB=LumA	>0.1
	HER2= Basal	>0.1
